# The D9N, N291S and S447X variants in the lipoprotein lipase (LPL) gene are not associated with Type III Hyperlipidemia

**DOI:** 10.1186/1471-2350-8-56

**Published:** 2007-08-29

**Authors:** David Evans, Frank U Beil

**Affiliations:** 1 Endokrinologie und Stoffwechsel, Medizinische Klinik III, Zentrum für Innere Medizin, Universitätsklinikum Hamburg-Eppendorf, Martinistrasse 52, 20246 Hamburg, Germany

## Abstract

**Background:**

Type III hyperlipidemia (Type III HLP) is associated with homozygosity for the ε2 allele of the APOE gene. However only about 10% of ε2 homozygotes develop Type III HLP and it is assumed that additional genetic and/or environmental factors are required for its development. Common variants in the LPL gene have been proposed as likely genetic co-factors.

**Methods:**

The frequency of the LPL SNPs D9N, N291S and S447X in 100 patients with hyperlipidemia and APOE2/2 genotype has been determined and compared to that in healthy blood donors and patients with hyperlipidemia.

**Results:**

There were no statistically significant difference in the frequencies of the variants between APOE2/2 patients and controls.

**Conclusion:**

It is unlikely that the D9N, N291S or S447X variants in the LPL gene play an important role in the development of Type III HLP.

## Background

Type III hyperlipidemia (Type III HLP, OMIM#107741) is a rare form of dyslipidemia characterised by elevated and approximately equal levels of plasma triglycerides and cholesterol and an increased risk for developing atherosclerosis [reviewed in [1,2]]. The great majority of sufferers are homozygous for the ε2 allele of the apolipoprotein E (APOE) gene. The remaining cases are the result of very rare mutations in the APOE gene giving rise to a dominant form of the disease and secondary factors play only a minor role [2]. Although APOE2/2 genotype is required for the development of Type III HLP only approximately 1–10% of APOE2/2 subjects suffer from the condition implying that additional genetic and/or environmental factors are necessary for its expression [1,2].

In a recent publication [3] we presented data which suggests that variants in the APOA5 gene constitute an important genetic co-factor for the development of TypeIII HLP. Since not all Type III HLP patients were carriers of the APOA5 SNPs it is probable that variants in other candidate genes are important in its development. Variants in genes coding for proteins involved in lipolysis such as lipoprotein lipase (LPL) have been suggested as possible co-factors [4,5]. Additional support for LPL as a candidate gene is provided by the suggestion that APOA5 acts as an activator of LPL activity in vivo [6].

A large number of studies have been performed on the association between three, functionally active single nucleotide polymorphisms (SNPs) in the LPL gene, namely D9N, N291S and S447X and hyperlipidemia [7-9]. The D9N (frequency in normal population 2–4%) and N291S (frequency in normal population 1–7%) have been associated in a meta-analysis with an increase in triglycerides of 20% and 31% respectively [9] and might therefore be expected to be present in increased frequency in patients with Type III HLP. The S447X SNP (frequency in normal population 17–22%) is associated with an 8% decrease in triglycerides and may thus be expected to be present at reduced frequency in patients with Type III HLP [9]. Further evidence that variation in the LPL gene may be a co-factor in the development of Type III HLP has been provided by Zhang et al [4] who reported that 4 out of 17 Type III HLP patients were carriers of the LPL N291S polymorphism compared with a frequency of 3 of 230 normal lipid controls. In a small study of eight Type III HLP patients, three were carriers of the N291S SNP neither the D9N nor the S447X were detected although a D9N carrier was detected in a group of three APOE2/2 normal controls [10]. Sijbrands et al [5] determined the frequencies of the D9N, N291S and S447X SNPs in the LPL gene in 49 probands (25 with Type III HLP) with APOE2/2 genotype. No carriers of the D9N SNP were detected but they found 3 carriers of the N291S and 2 of the S447X SNPs. As the authors comment, meaningful conclusions about the role of these variants cannot be drawn due to the low numbers and it is necessary to confirm their observations in a larger, independently recruited group of probands.

Over a ten year period we have assembled a group of 102 patients from the lipid clinic, UKE with APOE2/2 genotype and hyperlipidemia giving us the opportunity to reinvestigate the role of these LPL SNPs in the development of Type III HLP in a larger, independently recruited group of patients as suggested by Sijbrands et al [5]. We have compared the frequencies of the SNPs in hyperlipidemic patients with APOE2/2 genotype with healthy blood donors reasoning that if they play a role in the development of Type III HLP then their frequencies should be significantly different in patients compared to an unselected, healthy population. In addition the frequencies of the SNPs were determined in patients without APOE2/2 genotype attending the lipid clinic, Universitätsklinikum Hamburg-Eppendorf.

## Methods

### Patients

Patients who attended the lipid outpatient clinic, Universitätsklinikum Hamburg-Eppendorf (UKE) between 1997 and 2004 and 200 anonymous blood donors from the Blood Transfusion Service, UKE, were included in the study. Informed consent was obtained and the study was approved by the local ethics committee. This patient group has been previously described [3,11]. If clinically allowable, at the patients first visit to the clinic any existing lipid lowering medication is discontinued and dietary advice is given in a 60–90 minute discussion with a dietician. Six to eight weeks later the patient again attends the clinic and it is the lipid values obtained at this visit, i.e in the absence of lipid lowering therapy but after diet advice, that are used in this study. Probands were unrelated and reflect the multi-ethnic nature of the city of Hamburg and the surrounding area. The clinical characteristics of these patients are presented in Tables 1 and 2. There were 52 patients who were carriers of the R3500Q variant in the APOB gene who were diagnosed as familial defective APOB (FDB). An additional two carriers of the R3500Q variant had APOE2/2 genotype and so were excluded from the study. All patients with LDL above 330 mg/dl who were not carriers of the APOB variant were categorised as familial hypercholesterolemia (FH), this is sufficient for a diagnosis of FH using LDL levels alone according to the Dutch lipid clinics criteria [12].

**Table 1:** Clinical characteristics of the patients (excluding APOE2/2 genotype)

Male/Female	707/601
Age (years)	45.0 ± 13.8
BMI (kg/m^2^)	26.4 ± 6.4
Obese (BMI > 30)	258
CHD	168
DM2	104
Hypertriglyceridemia (TC ≤ 200 mg/dl, TG > 150 mg/dl)	89
Mixed hyperlipidemia (TC > 200 mg/dl, TG > 150 mg/dl)	601
Familial defective ApoB (R3500Q carrier)	52
Familial hypercholesterolemia (LDL > 330 mg/dl)	41
Hypercholesterolemia (TC > 200 mg/dl, TG ≤ 150 mg/dl)	434
Normal (TC ≤ 200 mg/dl, TG ≤ 150 mg/dl)	91
Total cholesterol (mg/dl)	242 ± 120
Triglycerides (mg/dl)	257 ± 405
LDL (mg/dl)	148 ± 96
HDL (mg/dl)	49 ± 18
ApoAI (mg/dl)	129 ± 56
ApoB (mg/dl)	127 ± 37
**APOE genotype**	
2/3	130
2/4	46
3/3	704
3/4	383
4/4	45
**LPL genotypes**	
*D9N*	
DD	1252
DN	55
NN	1
*N291S*	
NN	1234
NS	70
SS	4
*S447X*	
SS	1141
SX	155
XX	12

TC, total cholesterol; TG, triglycerides

**Table 2:** Clinical characteristics of the patients with APOE2/2 genotype

Male/Female	68/32
Age (years)	48.3 ± 11.0
BMI (kg/m^2^)	28.6 ± 4.2
Obese (BMI > 30)	32
CHD	17
DM2	10
ApoB (g/L)/TC (mmol/L) > 0.15	12
ApoB (g/L)/TC (mmol/L) < 0.15	88
Total cholesterol (mg/dl)	384 ± 134
Triglycerides (mg/dl)	587 ± 332
HDL (mg/dl)	43 ± 13
ApoAI (mg/dl)	140 ± 165
ApoB (mg/dl)	95 ± 40
**LPL genotypes**	
*D9N*	
DD	97
DN	3
NN	0
*N291S*	
NN	94
NS	6
SS	0
*S447X*	
SS	83
SX	16
XX	1

TC, total cholesterol

There were 41 patients in this group which probably represents an underestimate of the number of patients with FH since not all sufferers have such extremely high LDL levels. Since we have insufficient family data to allow a diagnosis in patients with LDL lower than 330 mg/dl, FH patients with LDL below this threshold are categorised as having hypercholesterolemia. Four hundred and thirty four patients had hypercholesterolemia (HChol), defined as total cholesterol (TC) above 200 mg/dl and triglycerides below 150 mg/dl. Six hundred and one patients presented with a mixed hyperlipidemia (mixed HLP) defined as total cholesterol above 200 mg/dl and triglycerides above 150 mg/dl. This diagnosis was preferred since there was not sufficient family data to allow a diagnosis of familial combined hyperlipidemia. Hypertriglyceridemia (triglycerides above 150 mg/dl and cholesterol below 200 mg/dl) was present in 89 patients and 91 probands with cholesterol below 200 mg/dl and triglycerides below 150 mg/dl were categorised as having normal lipids. There were 102 patients with APOE 2/2 genotype of whom two who were also carriers of the APOBR3500Q variant and so were not included in the study. Of the remaining 100 patients, 88 had Type III HLP based on the criteria of ApoB/total cholesterol ratio of below 0.15 as suggested by Blom et al [13].

### Biochemical measurements

Blood samples were taken after an overnight fast. Plasma cholesterol (TC) and triglycerides (TG) were determined using the GPO-PAP and CHOD-PAP kits respectively from Boehringer Mannheim. HDL was determined following precipitation of apo B containing lipoproteins with phosphotungstate (Boehringer Mannheim). LDL was estimated using the Friedwald formula. Apoliprotein AI (Apo AI) and Apo B were measured using the Beckmann Array 360 (Beckmann Instruments).

### Genotyping

DNA was isolated from 10 ml blood using the QIAamp DNA Blood Kit from Qiagen, Hilden, Germany. APOA5, APOE and LPL polymorphisms were determined as described [4,7,11,14].

### Statistical methods

Allele frequencies were determined by gene counting and compared using the chi-squared test. The affect of polymorphism on plasma lipids was analyzed using ANOVA with genotype as group variable and age, BMI, total cholesterol, triglycerides, HDL, LDL, apoB and apoAI as dependent variables. Where appropriate values were log transformed before analysis. A p value ≤ 0.05 was considered statistically significant. Analysis was performed using Statistica Software.

## Results

The incidence of the D9N, N291S and S447X SNPs in the LPL gene in the different patient groups and in the blood donors is presented in Tables 1, 2, 3. The frequency of carriers of the N allele of the D9N SNP in patients with APOE2/2 genotype (0.015) was not significantly different from either blood donors (0.03) or other patients from the lipid clinic (0.026). Similarly there were no significant differences in the frequency of carriers of the S allele of the N291S SNP, 0.03 in APOE2/2 patients, 0.018 in blood donors and 0.033 in lipid clinic patients. In the case of the S447X SNP, there were no significant differences in the frequency of the × allele, APOE2/2 patients 0.09, in blood donors, 0.085, and in lipid clinic patients, 0.068. Patients with APOE2/2 genotype were divided into those ApoB/total cholesterol ratio of below 0.15 (n = 88) i.e consistent with a diagnosis of Type III HLP and the remainder (n = 12). There was no statistically significant difference in the frequencies of any of the polymorphisms when the incidence between the blood donors, the lipid clinic and the patients with Type III HLP according to this criterion were compared (Table 3).

**Table 3:** Frequencies of LPL SNPs according to form of dyslipidemia

**D9N**	**n**	**DD**	**DN**	**NN**	**N Freq**
*APOE2/2 patients*					
ApoB (g/L)/TC (mmol/L) > 0.15	12	12	0	0	-
ApoB (g/L)/TC (mmol/L) < 0.15	88	85	3	0	0.015
*Lipid clinic patients*					
Hypertriglyceridemia (TC ≤ 200 mg/dl, TG > 150 mg/dl) n	89	82	7	0	0.039
Mixed hyperlipidemia (TC > 200 mg/dl, TG > 150 mg/dl) n	601	575	25	1	0.022
Familial defective ApoB (R3500Q carrier) n	52	50	2	0	0.019
Familial hypercholesterolemia (LDL > 330 mg/dl) n	41	38	3	0	0.036
Hypercholesterolemia (TC > 200 mg/dl, TG ≤ 150 mg/dl)	434	420	14	0	0.016
Normal (TC ≤ 200 mg/dl, TG ≤ 150 mg/dl) n	91	87	4	0	0.022
*Blood Donors*	200	188	12	0	0.03

**N291S**	**n**	**NN**	**NS**	**SS**	**S Freq**

*APOE2/2 patients*					
ApoB (g/L)/TC (mmol/L) > 0.15	12	11	1	0	0.041
ApoB (g/L)/TC (mmol/L) < 0.15	88	83	5	0	0.026
*Lipid clinic patients*					
Hypertriglyceridemia (TC ≤ 200 mg/dl, TG > 150 mg/dl) n	89	78	9	2	0.073
Mixed hyperlipidemia (TC > 200 mg/dl, TG > 150 mg/dl) n	601	560	39	2	0.036
Familial defective ApoB (R3500Q carrier) n	52	49	3	0	0.029
Familial hypercholesterolemia (LDL > 330 mg/dl) n	41	40	1	0	0.012
Hypercholesterolemia (TC > 200 mg/dl, TG ≤ 150 mg/dl)	434	421	13	0	0.015
Normal (TC ≤ 200 mg/dl, TG ≤ 150 mg/dl) n	91	86	5	0	0.027
*Blood Donors*	200	193	7	0	0.018

**S447X**	**n**	**SS**	**SX**	**XX**	**X Freq**

*APOE2/2 patients*					
ApoB (g/L)/TC (mmol/L) > 0.15	12	12	0	0	-
ApoB (g/L)/TC (mmol/L) < 0.15	88	71	16	1	0.092
*Lipid clinic patients*					
Hypertriglyceridemia (TC ≤ 200 mg/dl, TG > 150 mg/dl) n	89	84	5	0	0.028
Mixed hyperlipidemia (TC > 200 mg/dl, TG > 150 mg/dl) n	601	543	56	2	0.05
Familial defective ApoB (R3500Q carrier) n	52	43	9	0	0.087
Familial hypercholesterolemia (LDL > 330 mg/dl) n	41	34	6	1	0.096
Hypercholesterolemia (TC > 200 mg/dl, TG ≤ 150 mg/dl)	434	357	69	8	0.098
Normal (TC ≤ 200 mg/dl, TG ≤ 150 mg/dl) n	91	80	10	1	0.066
*Blood Donors*	200	166	34	0	0.085

TC, total cholesterol; TG, triglycerides

Patients who did not have APOE2/2 genotype were grouped according to the form of hyperlipidemia (see Materials and Methods) and the frequencies of the LPL SNPs compared. Consistent with reports in the literature the frequency of the N291S SNP was higher and that of the S447X lower in patients with either hypertriglyceridemia or mixed hyperlipidemia compared to either patients with normal lipids or the healthy blood donor population controls. The frequency of the S allele of the N291S determined when mixed hyperlipidemia and hypertriglyceridemia patients were combined, 0.04, was significantly higher than in the normal lipid controls (i.e combined blood donor and patients with normal lipids), 0.02, p = 0.03. The frequency of the × allele of the S447X was significantly lower, 0.047 compared to 0.078, p = 0.005. There was no significant difference in the frequency of the D9N polymorphism between patients with elevated triglycerides and controls. Median triglycerides were higher in carriers of the D9N variant, 178 mg/dl compared to 144 mg/dl, p = 0.02, and the N291S variant, 179 mg/dl compared to 144 mg/dl, p = 0.03 and lower in carriers of the S447X variant, 126 mg/dl compared to 171 mg/dl, p = 0.00004.

The frequency of the S allele of the N291S in APOE2/2 patients with Type III HLP, 0.026, lay between that of the patients with elevated triglycerides, 0.04, and controls, 0.02, and in no case was there a statistically significant difference. The frequencies of the N allele of the D9N SNP in patients, with Type III HLP, 0.015 were not significantly different from those for patients with elevated triglycerides, 0.025 or controls, 0.027. There was no significant difference between the frequency if the × allele of the S447X SNP in APOE2/2 patients, 0.09, and controls, 0.078, whereas the frequency in patients with elevated triglycerides was significantly lower, 0.047, p = 0.01. In Table 4 are presented the LPL genotype frequencies according to APOE genotype. The frequency of double carriers does not exceed that expected by chance.

**Table 4:** LPL and APOE genotypes

**LPL**				**APOE**					
**S447X**	**D9N**	**N291**	**n**	**2/2**	**2/3**	**2/4**	**3/3**	**3/4**	**4/4**

SS	DD	NN	1095	76	96	41	548	295	39
		NS	70	6	9	1	32	20	2
		SS	4	0	1	0	2	1	0
	DN	NN	53	1	7	2	29	13	1
		NS	1	0	0	0	1	0	0
	NN	NN	1	0	0	0	1	0	0
SX	DD	NN	163	14	17	2	78	49	3
		NS	4	0	0	0	2	2	0
	DN	NN	4	2	0	0	1	1	0
XX	DD	NN	12	1	0	0	10	1	0
		NS	1	0	0	0	0	1	0
Total			1408	100	130	46	704	383	45

Previously we reported that in these patients carriers of the -1131T > C and S19W variants in the APOA5 gene were more frequent in patients with Type III HLP than in controls [3]. Of the three APOE2/2 patients who were carriers of the D9N variant, two had APOA5 genotype TT/SS and the other, TT/SW. The APOA5 genotypes of the N291S carriers, were three TT/SS, two TC/SS and a single TT/SW. Ten of the S447X carriers were APOA5 TT/SS, four TT/SW, two TC/SS and one was TC/SW. Thus APOE2/2 patients who were carriers of the APOA5 variants were neither more nor less likely to be carriers of a LPL SNP than non-carriers.

## Discussion

Although the majority of patients with Type III HLP have APOE genotype 2/2 only a minority of APOE2/2 subjects develop Type III HLP implying additional genetic and/or environmental factors are required [1,2]. The hypothesis we wished to test in this study is that common variants in the LPL gene may be among such additional genetic factors. This hypothesis was based on the report of Zhang et al [4] who found a significantly increased frequency of the N291S SNP in Type III HLP patients plus a large number of reports in which the N291S and D9N SNPs were associated with increased triglycerides and familial combined hyperlipidemia whereas the S447X SNP has a lower frequency in such patients [9]. If these variants are important co-factors for the development of Type III HLP we would expect to observe a significantly increased frequency of the N291S and D9N SNPs and a decrease in the frequency of the S447X SNP in patients compared to controls.

Since we failed to observe significantly altered frequencies in any of the LPL SNPs in APOE2/2 patients compared to controls, even under the more stringent criteria of an apoB/total cholesterol ratio below 0.15, we conclude that it is unlikely that these SNPs play a significant role in the development of Type III HLP. This is in contrast to the conclusions of Zhang et al [4] who reported for the S allele of the N291S of 0.16 (4 carriers amongst 17 patients) in Type III HLP compared to 0.006 (3/230) in controls. Sijbrands et al. [5] detected 2 carriers among 25 patients with Type III HLP but they also report a proband with APOE2/2 genotype who was a carrier of N291S but who did not have Type III HLP. In their study Sijbrands et al [5] did not detect APOE2/2 probands who were carriers of the D9N SNP, Zhang et al [4] did not investigate this variant.

As far as we are aware this is the first report investigating the S447X SNP and Type III HLP. Since the S447X SNP is associated with lower triglycerides it might be assumed that it could act as a protective variant and reduce the chance of carriers with APOE2/2 genotype from developing Type III HLP and therefore be present at a reduced frequency in these patients, similarly to that observed amongst lipid clinic patients with elevated triglycerides. Our finding that the frequency of the S447X is similar in Type III HLP patients as in controls shows that this is not the case. Although our data suggest that common variants in the LPL gene are not an important factor in the development of Type III HLP at a population level they may nevertheless influence the development of the condition in APOE2/2 individuals who are carriers.

In our patient group the frequency of the N291S SNP was increased and the S447X decreased in non-APOE2/2 patients with elevated triglycerides compared to controls as reported in a number of studies [9], however we did not see a significant association with the D9N SNP, the variant with the least reported effect on triglycerides [9]. Since LPL SNPs are associated with triglycerides in our patients who are not APOE2/2 this implies that the triglyceride raising effect of the N291S variant, 30%, and the lowering effect of the S447X variant, 8%, are masked by the other genetic and environmental factors contributing to the development of Type III HLP in APOE2/2 patients. Consistent with this point, we observed no statistically significant differences in the plasma lipid values of APOE2/2 subjects who were carriers of LPL variants and those who were not whereas for non APOE2/2 subjects the D9N and N291S were associated with increased and the S447X with decreased triglycerides.

The contrast between these findings and those of Zhang et al [4] is probably due to the greater number of probands in our study. In an earlier, small study we also reported a high frequency of the N291S SNP in Type III HLP patients [10]. The significance in the Zhang study is also magnified by the low frequency in the controls, 0.007. A weakness of our study is the relatively small number of patients, this is due to the rareness of Type III HLP. The frequency of APOE2/2 genotype in normal population is approximately 1% of whom 1–10% develop Type III HLP [1,2] giving a frequency of 1–10 in 10,000 and therefore 100 probands represents the Type III HLP cases in a population of 100.000–1.000.000, therefore in the context of Type III HLP this is a relatively large study.

The role of multiple rare alleles in the LPL gene and the development of Type III HLP remains to be investigated. A high frequency of multiple rare variants in the ABCA1 gene has been reported in patients with low HDL [15]. Assuming a frequency of LPL deficiency of one in a million, then a frequency of obligate heterozygotes for mutation in the LPL gene of 0.2% is to be expected and so it cannot be ruled out that individually rare mutations in the LPL gene collectively play a role in the development of Type III HLP in APOE2/2 individuals.

## Conclusion

In this, to our knowledge the largest study of its kind performed to date, we could not confirm the previously reported significantly increased incidence of the N291S SNP in LPL in Type III HLP patients. In addition we found no association between the D9N and S447X SNPs and Type III HLP and so we conclude that these three common variants in the LPL gene are not an important factor in the development of Type III HLP in probands with APOE2/2 genotype.

## Competing interests

The author(s) declare that they have no competing interests.

## Authors' contributions

DE carried out the molecular genetic studies, performed the statistical analysis, and wrote the manuscript. FB participated in the design of the study, was responsible for the clinical aspects and read and approved the final manuscript.

## Pre-publication history

The pre-publication history for this paper can be accessed here:


